# The Gut Microbiota Profile According to Glycemic Control in Type 1 Diabetes Patients Treated with Personal Insulin Pumps

**DOI:** 10.3390/microorganisms9010155

**Published:** 2021-01-12

**Authors:** Sandra Mrozinska, Przemysław Kapusta, Tomasz Gosiewski, Agnieszka Sroka-Oleksiak, Agnieszka H. Ludwig-Słomczyńska, Bartłomiej Matejko, Beata Kiec-Wilk, Malgorzata Bulanda, Maciej T. Malecki, Pawel P. Wolkow, Tomasz Klupa

**Affiliations:** 1 Jagiellonian University Medical College, Faculty of Medicine, Department of Metabolic Diseases, 2 Jakubowskiego Street, 30-688 Krakow, Poland; rubita6@o2.pl (S.M.); b.matejko@uj.edu.pl (B.M.); beata.kiec-wilk@uj.edu.pl (B.K.-W.); maciej.malecki@uj.edu.pl (M.T.M.); 2 University Hospital, Department of Metabolic Diseases, 2 Jakubowskiego Street, 30-688 Krakow, Poland; 3 Jagiellonian University Medical College, Center for Medical Genomics OMICRON, 7c Kopernika Street, 31-034 Krakow, Poland; przemyslaw.kapusta@uj.edu.pl (P.K.); agnieszka.ludwig@uj.edu.pl (A.H.L.-S.); pawel.wolkow@uj.edu.pl (P.P.W.); 4 Jagiellonian University Medical College, Faculty of Medicine, Department of Microbiology, 18 Czysta Street, 31-121 Krakow, Poland; tomasz.gosiewski@uj.edu.pl (T.G.); agnieszka.sroka@uj.edu.pl (A.S.-O.); malgorzata.bulanda@uj.edu.pl (M.B.)

**Keywords:** KEGG pathways, microbiota, next-generation sequencing, type 1 diabetes

## Abstract

Recently, several studies explored associations between type 1 diabetes (T1DM) and microbiota. The aim of our study was to assess the colonic microbiota structure according to the metabolic control in T1DM patients treated with insulin pumps. We studied 89 T1DM patients (50.6% women) at the median age of 25 (IQR, 22–29) years. Pielou’s evenness (*p* = 0.02), and Shannon’s (*p* = 0.04) and Simpson’s diversity indexes (*p* = 0.01), were higher in patients with glycosylated hemoglobin (HbA1c) ≥ 53 mmol/mol (7%). There were no differences in beta diversity between groups. A linear discriminant analysis effect size (LEfSe) algorithm showed that one family (*Ruminococcaceae*) was enriched in patients with HbA1c < 53 mmol/mol, whereas one family (*Streptococcaceae*) and four species (*Ruminococcus torques*, unclassified species of *Lactococcus*, *Eubacteroim dolichum*, and *Coprobacillus cateniformis*) were enriched in patients with HbA1c ≥ 53 mmol/mol. We found that at class level, the following pathways according to Kyoto Encyclopedia of Genes and Genomes were enriched in patients with HbA1c < 53 mmol/mol: bacterial motility proteins, secretion system, bacterial secretion system, ribosome biogenesis, translation proteins, and lipid biosynthesis, whereas in patients with HbA1c ≥ 53 mmol/mol, the galactose metabolism, oxidative phosphorylation, phosphotransferase system, fructose, and mannose metabolism were enriched. Observed differences in alpha diversity, metabolic pathways, and associations between bacteria and HbA1c in colonic flora need further investigation.

## 1. Introduction

Type 1 diabetes mellitus (T1DM) is an autoimmune disease that results from the destruction of the pancreatic beta cells producing insulin [1]. Because of that, patients with T1DM require lifelong insulin therapy that mimics the basal and mealtime release of insulin by the pancreas [1]. Intensive insulin therapy involves multiple insulin injections (MDI) or continuous subcutaneous insulin infusion (CSII) [1].

The pathogenesis of T1DM is not fully understood [1]. The genetic–specific HLA predisposition, and epigenetic and environmental factors such as diet or viral infections, are reported to contribute to the development of the disease [1,2]. It has been postulated that one of the potential factors contributing to the development of T1DM or triggering the disease could be dysbiosis of interstitial bacterial flora [3,4]. The underlying mechanisms are unknown. It has been proposed, for instance, that alternations in gut microbiota can cause a decrease in bacteria producing butyrate, a short chain fatty acid (SCFA), and as a consequence, can cause an increase in permeability and autoimmunity for T1DM [5]. Recently, several studies have shown the relationships between beta-cell autoimmunity, occurrence of T1DM, and microbial dysbiosis [6,7,8,9,10,11,12,13,14].

The potential association between gut dysbiosis and T1DM may be, however, not only casual, but it could potentially affect glycemic control in the already developed disease. Metabolic endotoxemia is described as a condition without acute infection when there is an elevated lipopolysaccharide (LPS) level, a main component of the Gram negative bacteria membrane [15]. Increased fasting and postprandial LPS levels are observed in both T1DM and T2DM [15]. Some data, mostly based on type 2 diabetes mellitus (T2DM), indicate that derangements in the composition of the microbiota may play a role in the development of “metabolic endotoxemia” leading to hyperglycemia based on immune independent mechanisms [16,17].

A recent meta-analysis has shown that the modification of gut microbiome following probiotics usage may improve glycemic status in T2DM [18]. Since those mechanisms and interventions could be also applicable to T1DM, one could speculate that there could also be a relationship between gut microbiota structure and glycemic status in T1DM individuals.

It has been reported that probiotics/prebiotics can decrease glucose level, increase SCFAs, diminish the inflammatory pathway, and reduced systematic endotoxemia in T1DM animal studies [2]. It is hypothesized that prebiotics via changing intestinal microbiota could lead to better glycemic control in T1DM patients [19].

Given reported improvement in glucose control after changes in microbiota in T1DM animal studies, we hypothesized that colonic flora differs according to the metabolic control in T1DM patients.

The aim of our study was to compare gut microbiota structure and their metabolic pathways between T1DM patients meeting the current criteria of glycemic control [20,21] with glycosylated hemoglobin (HbA1c) below 53 mmol/mol (7%) and patients with HbA1c equal to or greater than 53 mmol/mol (7%).

## 2. Materials and Methods

### 2.1. Study Setting and Eligibility

The study was performed from 2016 to 2018. Ninety-four consecutive patients with T1DM treated with insulin pumps at our Outpatients Diabetic Clinic were enrolled.

Since the main goal of the study was to assess the relationship between microbiota structure and HbA1c per se, we aimed to perform our analysis on as homogenous a group as possible.

Patients with T1DM who use an insulin pump and confirmed readiness to cooperate with the research center were eligible for the study.

The exclusion criteria were chosen carefully and included clinical conditions and patients’ behavior, which could have a significant impact on microbiota status irrespective of glycemic control. These included the following:

- confirmed infection of the gastrointestinal tract or using probiotics or taking antibiotics for up to 30 days before delivery of a stool sample,
- chronic inflammatory bowel disease of unknown etiology, active cancer (especially of the gastrointestinal tract),
- immunodeficiency,
- the presence of advanced late complications of diabetes, and
- low and very high carbohydrate consumption defined as below 100 or over 400 g of carbohydrates verified by questionnaire and insulin pump downloads.

The study received approval from the Jagiellonian University Ethics Committee (number KBET/256/B/2014, date 27.11.2014). All participants provided written informed consent in accordance with the Declaration of Helsinki.

### 2.2. Study Investigations

We obtained reports from personal insulin pumps (14 days). The reports provided the information about average glucose level along with standard deviation (SD); basal, bolus, and total daily insulin dose; the amount of glucose measurements per day; and the average amount of carbohydrates eaten per day. The patients were asked to complete the survey regarding eating habits. HbA1c was assessed (high-performance liquid chromatography using the Variant II Turbo analyzer, Hercules, CA, USA, or HbA1c, Quo Lab HbA1c, EKF Diagnostics) and stool samples were collected from all patients. 

### 2.3. DNA Isolation and 16S Metagenomic Sequencing

Bacterial DNA was isolated using Genomic Mini AX Stool Spin (A&A Biotechnology, Gdynia, Poland), with several modifications, as described in our previous publication [22].

Libraries were prepared according to the Illumina 16S Metagenomic Sequencing Library Preparation protocol. After that, the libraries at a concentration 10 pM with 20% PhiX spike-in control were sequenced on Illumina MiSeq (Illumina, Inc., San Diego, CA, USA) using the V3 sequencing kit (300 bp paired-end reads) [22].

### 2.4. Sequencing Data Analysis

Samples were processed and analyzed using the Quantitative Insights Into Microbial Ecology 2 (QIIME2, version 2019.7) [23] custom pipeline. Briefly, the quality of demultiplexed paired-end reads from MiSeq (2 × 300 bp) was evaluated and the reads were trimmed to remove primers and poor-quality bases with cutadapt [24]. Trimmed pair-end sequences were denoized and merged with DADA2 [25] to generate amplicon sequence variants (ASVs). Next, reference-based chimera filtering was queried against the reference database (Greengenes version 13.8) at 99% similarity with vsearch [26].

Low-abundance ASVs were eliminated when they appeared in less than three samples or the number of counts across all samples was <5. The SATé-enabled phylogenetic placement (SEPP) algorithm was used to build the tree for phylogenetic diversity computation [27]. QIIME2 diversity core-metrics-phylogenetic analyses were used to compute alpha and beta diversity values, and rarefaction curve analysis was used to estimate the completeness of microbial community sampling. We also computed default alpha and beta diversity metrics and generated principal coordinates analysis (PCoA) plots for each of the beta diversity metrics using the EMPeror [28]. The alpha and beta diversity indices of the groups were compared using QIIME2 longitudinal pairwise-differences plugin using the *t*-test. Correlations with alpha and beta diversity indices were calculated with the QIIME2 plugins using Spearman correlation. Generated ASVs were assigned to taxonomy using a naive Bayes classifier [29] that was pre-trained on the v3-v4 rRNA regions in Greengenes version 13.8, at 99% similarity. The bacterial composition was analyzed at phylum, class, order, family, genus, and species levels. Differential abundance between groups at each taxonomic level was tested using analysis of the composition of microbiomes (ANCOM) [30]. Additionally, we used ‘MaAsLin2′ package [31] in R statistical environment to verify the multivariable association between clinical metadata and relative abundance of taxa at different taxonomical levels. The regression coefficient (coef) in MaAsLin2 is the effect size, which represents the rate of change in abundance of taxa per 1 score of measurements. Both, ANCOM and MaAsLin2 control for FDR was at level 0.05. To find taxa and pathways that explain differences between our group, we used LEfSe (Linear Discriminant Analysis Effect Size) [32], which is an algorithm for high-dimensional biomarker discovery and a tool to identify genomic features, such as taxa or pathways. The LEfSe was used with the default parameters (*p* < 0.05 and Linear Discriminant Analysis [LDA] score >2.0). First, we performed differential abundance on species taxonomical level, and then we predicted KEGG (Kyoto Encyclopedia of Genes and Genomes) [33] based on functional profiles using PICRUSt (Phylogenetic Investigation of Communities by Reconstruction of Unobserved States) software v.1.1.4 [34]. Since PICRUSTt requires an operational taxonomic unit (OTU)_ table ‘closed-reference’ picked against Greengenes database, we clustered our ASVs sequences with Greengenes 13.8 database, at 99% similarity, using the vsearch cluster-features-closed-reference [26] plugin in QIIME2 [23].

### 2.5. Statistical Analysis

Continuous variables are expressed as median (interquartile range (IQR)) or mean (± SD). Categorical variables are presented as count (percentages). The normality of continuous variables was verified using the Shapiro–Wilk test. The equality of variances was checked by the Levene’s test. Groups were compared using Student’s *t*-test, Welch *t*-test, Mann–Whitney U test, and Fisher’s exact test, appropriately. Calculations were performed using Statistica 13 software (StatSoft Inc., Tulsa, OK, USA) and package R ver 3.6.2 [35].

## 3. Results

### 3.1. Study Population

From all 94 stool samples, 5 samples were excluded due to low read count after DADA2 denoising, chimera removal, and filtering. Therefore, the final study population consisted of 89 patients (50.6% women). The median age of the cohort was 25 (22–29) years. The patients were divided into two groups according to the HbA1c level: patients with HbA1c below 53 mmol/mol (7%) (*n* = 43) and patients with HbA1c equal to or greater than 53 mmol/mol (7%) (*n* = 46). There were no differences in age, body mass index (BMI), and duration of diabetes between both groups (Table 1).

Patients with HbA1c below 53 mmol/mol (7%) more often measured their glucose level and declared higher consumptions of carbohydrates per day. There were no differences in daily insulin dose or in dose of insulin per kilogram body weight between groups (Table 1).

Twenty patients suffered from hypothyroidism, two from celiac disease, two from vitiligo, one patient had autoimmune hepatitis, one patient had Addison–Biermer anemia, and four patients had hypertension (Table 1). The groups did not differ in hypothyroidism diagnosis. The groups did not differ in general eating habits (Table 2).

### 3.2. 16S rRNA Sequencing Analysis

After DADA2 denoising, chimera removal, and filtering of 94 patients, 5 samples were excluded due to low read count. Sequencing analysis for the 89 patients in the final cohort provided a median of 14,865 (IQR, 11,067–17,897). The best sample contained 33,933 reads, while the worst contained 6267 reads. DADA2 pipeline resulted in 480, features (ASVs), with total frequency of 1,340,777 features. Based on rarefaction curves, the diversity analysis was calculated with a minimum number of 6267 sequences per samples, which corresponded to the minimum frequency.

### 3.3. Diversity Analysis

We first explored the relationship between microbial alpha diversity (Shannon’s diversity index, observed OTUs, Faith’s Phylogenetic Diversity and Pielou’s evenness, Simpson’s diversity index, and Chao index) in the low (< 53 mmol/mol (7%)) and high (≥ 53 mmol/mol (7%)) HbA1c level groups. Only Shannon’s diversity index (*p* = 0.04), Pielou’s evenness (*p* = 0.02), and Simpson’s diversity index (*p* = 0.01), but not observed OTUs (*p* = 0.34), Faith’s Phylogenetic Diversity (*p* = 0.9), and Chao index (*p* = 0.33), were higher in the HbA1c high group (≥ 53 mmol/mol (7%)).

**Figure 1:**
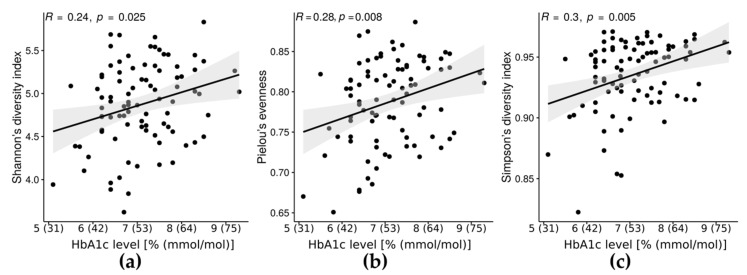
The relationships between glycosylated hemoglobin (HbA1c) level [% (mmol/mol)] and Shannon’s diversity index (**a**), Pielou’s evenness (**b**) and Simpson’s diversity index (**c**).

Furthermore, HbA1c level had a positive correlation with Shannon’s diversity index (R = 0.24, *p* = 0.025), Pielou’s evenness (R = 0.28, *p* = 0.008), and Simpson’s diversity index (R = 0.3, *p* = 0.005, Figure 1). 

Average glucose level was correlated with Pielou’s evenness (R = 0.26, *p* = 0.02) and Simpson’s diversity index (R = 0.27, *p* = 0.01). Standard deviation of average glucose level was not correlated with any analyzed measure of alpha diversity. Both average glucose level and standard deviation of average glucose level were associated with HbA1c (R = 0.63, *p* <0.001 and R = 0.55, *p* <0.001, respectively). Dose of insulin per kilogram body weight was negatively associated with Pielou’s evenness (R = −0.27, *p* = 0.015) and Simpson’s diversity index (R = −0.23, *p* = 0.03).

**Figure 2:**
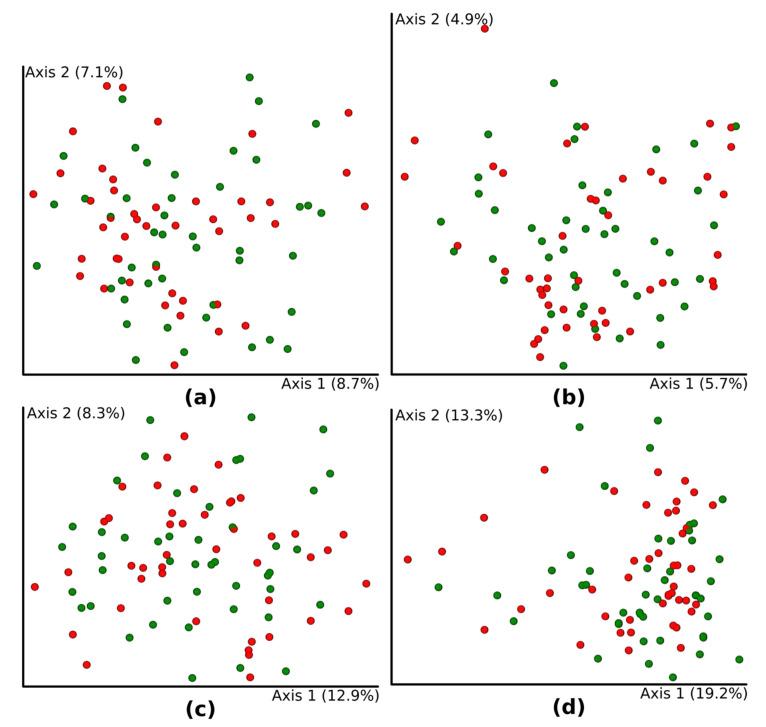
Principal coordinates analysis PCoA plots of beta diversity of patients with type 1 diabetes (T1DM) and with glycosylated hemoglobin (HbA1c) below 53 mmol/mol (7%) (green dots) and T1DM patients with HbA1c equal to or greater than 53 mmol/mol (7%) (red dots). Differences were presented as Bray–Curtis dissimilarity (*p* = 0.18) (**a**), Jaccard distance (*p* = 0.26) (**b**), unweighted UniFrac (*p* = 0.59) (**c**), and weighted UniFrac (*p* = 0.33) (**d**).

HbA1c groups did not differ in four beta diversity indices: Bray–Curtis dissimilarity (*p* = 0.18), Jaccard distance (*p* = 0.26), and unweighted (*p* = 0.59) and weighted UniFrac (*p* = 0.33). Moreover, patients were not separated or clustered according to Principal Coordinates Analysis (PCoA) of beta diversity metrics (Figure 2).

HbA1c level (as a continuous variable) was not associated with beta diversity indices, Bray–Curtis dissimilarity (R = 0.03, *p* = 0.48), Jaccard distance (R = 0.04, *p* = 0.39), or unweighted (R = 0.05, *p* = 0.28) and weighted UniFrac (R = 0.05, *p* = 0.32). Average glucose level, standard deviation of average glucose level, and insulin per kilogram body weight were also not correlated with any of beta diversity indices (data not shown).

#### 3.3.1. Bacterial Profile

After annotation of 480 ASVs with Greengenes taxonomy, at 99% similarity, we were able to classify 100% ASVs at the phylum (L2) and at class (L3), 99.8% at order (L4), 90.2% at family (L5), 66.2% at genus (L6), and 27.7% at species (L7) level. A total of 135 species-level taxa were identified, representing 9 phyla, 14 classes, 18 orders, 41 families, and 86 genera.

**Figure 3:**
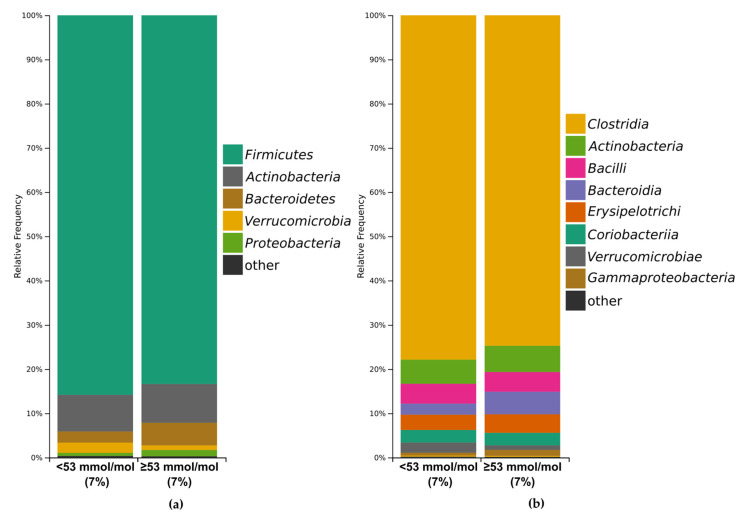
Relative abundance of most common bacterial phyla (**a**) and classes (**b**) at each group. All taxa with abundance below 1% are represented as ‘other.’

There were the following baseline bacterial profiles with an abundance of >1% at the phylum level in patients with HbA1c below 53 mmol/mol (7%) and patients with HbA1c ≥ 53 mmol/mol (7%): *Firmicutes* (85.8% versus (vs.) 83.3%), *Actinobacteria* (8.3% vs. 8.8%), *Bacterioidetes* (2.5% vs. 5.1%), *Verrucomicrobia* (2.3% vs. 1.0%), *Proteobacteria* (0.7% vs. 1.4%), and ‘other’ (0.4% vs. 0.4%; Figure 3a). At the class level, baseline bacterial profiles included *Clostridia* (77.9% vs. 74.7%), *Actinobacteria* (5.4% vs. 5.9%), *Bacilli* (4.5% vs. 4.4%), *Bacteroidia* (2.5% vs. 5.1%), *Erysipelotrichi* (3.4% vs. 4.2%), *Coriobacteriia* (2.8% vs. 2.8%), *Verrucomicrobiae* (2.3% vs. 1.0 %), *Gammaproteobacteria* (0.6 % vs. 1.4 %), and ‘other’ (0.6% vs. 0.5%; Figure 3b), respectively.

Core microbiome analysis at species level showed that both subgroups shared 129 taxa, three were unique to patients with HbA1c < 53 mmol/mol (7%) and five were unique to subjects with HbA1c ≥ 53 mmol/mol (7%). Detailed taxonomic information about overlaps between groups is available in supplementary data (Supplement 1).

The groups did not differ in *Firmicutes/Bacteroidia* (*F/B*) ratio (*p* = 0.59). We observed borderline significance in *F/B* ratio between men and women (*p* = 0.08), although there were no differences in the *F/B* ratio between HbA1c groups in females (*p* = 0.39), and in men (*p* = 0.98). The *F/B* ratio was not correlated with HbA1c (*p* = 0.33) or mean glucose level (*p* = 0.28). There was no association between *F/B* ratio and dose of insulin per kilogram in patients adjusted for HbA1c (*p* = 0.77) or mean glucose level (*p* = 0.78).

#### 3.3.2. Differential Abundance of Microbial Taxa

**Figure 4:**
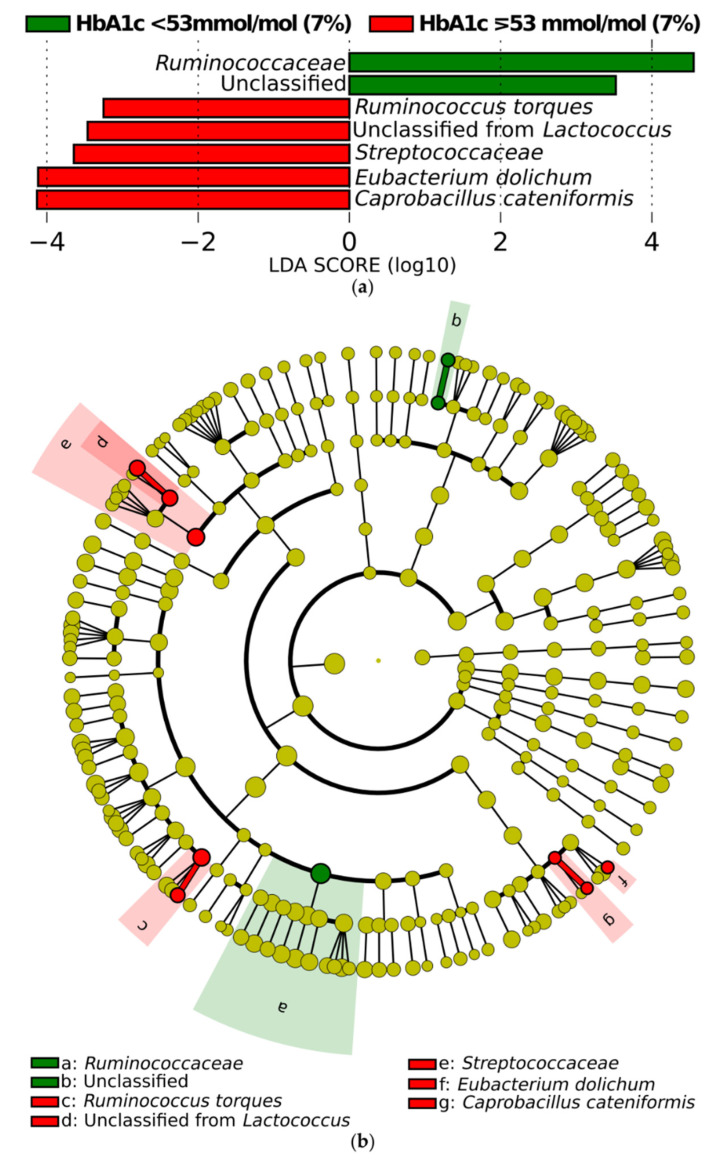
The linear discriminant analysis effect size (LEfSe) method showed the significantly different taxa of patients with type 1 diabetes (T1DM) associated with glycosylated hemoglobin (HbA1c) < 53 mmol/mol (7%) (green) and ≥ 53 mmol/mol (7%) (red). The taxa with significantly different abundances among the groups were presented as barplot (**a**) with linear discriminant analysis (LDA) score, and on the clarogram (**b**). From inside to outside, the dots denote the kingdom, phylum, class, order, family, genus, and species.

There were no differences in ANCOM results for phylum, class, order, family, genus, or species between both groups. When adjusted for sex, age, BMI, diabetes duration in years, and total daily insulin in units, still no differences on tested taxonomical level were found between both HbA1c groups. Then, we decided to use MaAsLin2 to verify the multivariable association between clinical metadata, such as sex, age, BMI, diabetes duration in years, total daily insulin, daily carbs, and HbA1c in a linear model. We performed analysis on phylum, class, order, family, genus, and species level, but did not find any significant association (all FDR > 0.05) between tested clinical data and relative abundance of gut microbiota in T1DM patients. Since there were no differences in differential abundance, the changes in the microbial community may be more discrete; therefore, we performed additional analysis with the LEfSe algorithm to determine the bacteria that might be associated with differences in HbA1c (Figure 4). In patients with HbA1c < 53 mmol/mol (7%), one family (*Ruminococcaceae*) was enriched. One family (*Streptococcaceae*) and four species (*Ruminococcus torques,* unclassified species of *Lactococcus, Eubacteroim dolichum,* and *Coprobacillus cateniformis*) were enriched in patients with HbA1c ≥ 53 mmol/mol (7%).

Additionally, in MaAsLin2 HbA1c (as a continuous variable), average glucose level or standard deviation of average glucose level were not correlated with any OTU at phylum, class, order, family, genus, or species level. The dose of insulin per kilogram body weight was positively correlated with *Bacteroides uniformis* (coef = 0.008, FDR = 0.037), and we found that insulin per kilogram body weight after adjustment for age, sex, and standard deviation of average glucose level was negatively correlated with phyla *Firmicutes* (coef= −0.017, FDR = 0.043).

#### 3.3.3. Functional Profiles of Gut Microbiota

**Figure 5:**
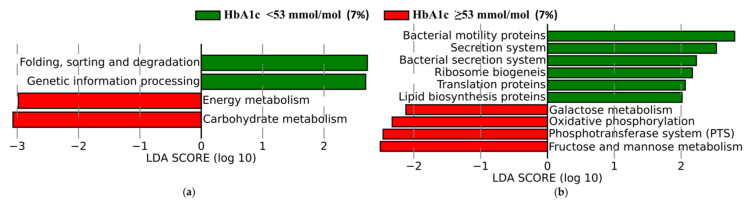
Functional divergence between the faecal microbiota of patients with type 1 diabetes and glycosylated hemoglobin (HbA1c) < 53 mmol/mol (7%) and ≥ 53 mmol/mol (7%). The comparisons of pathways at level 2 (phylum) (**a**) and level 3 (class) (**b**) were performed based on Kyoto Encyclopedia of Genes and Genomes.

Using LEfSe, we also explored KEGG pathways that were differentially enriched between HbA1c < 53 mmol/mol (7%) and HbA1c ≥ 53 mmol/mol (7%). At phylum level, we observed only four pathways, and interestingly, the group with higher HbA1c was enriched in the pathway associated with Energy Metabolism and Carbohydrate Metabolism (Figure 5a). At class level, we observed 10 metabolic pathways differentially enriched between patients in our groups (Figure 5b). In patients with HbA1c < 53 mmol/mol (7%) the following pathways were enriched: bacterial motility proteins, secretion system, bacterial secretion system, ribosome biogenesis, and translation proteins lipid biosynthesis, whereas in patients with HbA1c ≥ 53 mmol/mol (7%), the galactose metabolism, oxidative phosphorylation, phosphotransferase system, fructose, and mannose metabolism were enriched.

## 4. Discussion

To our knowledge, we report for the first time differentially enriched KEGG metabolic pathways according to HbA1c among a group of patients with T1DM in the Polish cohort.

Surprisingly, in our study, we noticed slightly greater alpha diversity in T1DM patients with HbA1c ≥ 53 mmol/mol (7%) compared to patients with T1DM and <53 mmol/mol (7%). We also found a positive correlation between alpha diversity and HbA1c in the T1DM cohort. Moreover, we observed some significantly different taxa using the LEfSe algorithm between groups. Our study demonstrates differences in colon microbiota regarding the level of metabolic control of diabetes in T1DM patients.

First of all, we would like to underline that our cohort of young adults with T1DM was well-characterized and homogenous. All patients were treated with personal insulin pumps, with downloading of the devices allowed to obtain precise information concerning insulin dosing. Study participants were free from advanced complications of diabetes, quite homogenous with regard to diet, and the majority of them were free from comorbidities that could affect study results (e.g., celiac disease, *n* = 2). This minimizes the effect of confounding factors on the relationship of single variable, HbA1c, and the microbiota profile.

In our study, we did not observe differences in ANCOM results for phylum, class, order, family, genus, or species between individuals with HbA1c level below 53 mmol/mol (7%) and individuals with HbA1c equal to or greater than 53 mmol/mol (7%). However, we noticed differences between groups in some alpha diversity indices. We observed higher alpha diversity in patients with higher HbA1c and a positive correlation between HbA1c and alpha diversity. A previous study showed a decrease in alpha diversity in patients after the seroconversion and before T1DM development, and the decrease in alpha diversity was also observed between T1DM patients in comparison to controls [36]. Also, patients with newly diagnosed T2DM had decreased diversity compared to healthy control group [37]. There are inconclusive results whether obesity is associated with changes in alpha diversity [38], as an increased diversity in obese patients compared to controls was also reported [39].

We did not find differences in *F/B* ratio between our two groups of patients. In some previous cohort studies, it has been demonstrated that patients with T1DM had a decreased *F/B* ratio [40,41]. However, we found that insulin per kilogram body weight after adjustment for age, sex, and standard deviation of average glucose level was negatively correlated with phyla *Firmicutes.* That could indicate the potential role of hipo/hyperinsulinemia itself on microbiota composition.

We did not notice any correlation between HbA1c level and single microbal OTU. We found that the family *Ruminococcaceae* was enriched in a group of patients with HbA1c < 53 mmol/mol (7%) based on LEfSe algorithm, which could be partially in agreement with the a study of Hang et al., who observed negative correlation between HbA1c and both *Ruminococcaceae* and its genus *Faecalibacterium* [40]. Previously, Qi et al. observed a positive correlation between relative abundance of *Blautia* and HbA1c among newly diagnosed children with T1DM and healthy controls [11], and Fassatoui et al. noticed a negative correlation between HbA1c and amount of *Akkermansia muciniphia* [41].

As we did not perform the shotgun sequencing, we predicted KEGG pathways based on functional profiles using PICRUSt. We found that microbiota profile in patients with HbA1c < 53 mmol/mol (7%) were characterized by more abundant metabolic pathways related to bacterial motility proteins, secretion system, bacterial secretion system, ribosome biogenesis, translation proteins, and lipid biosynthesis proteins, whereas in patients with HbA1c ≥ 53 mmol/mol (7%), they were related to the galactose metabolism, oxidative phosphorylation, phosphotransferase system, fructose, and mannose metabolism. The lipid biosynthesis proteins include the acetyl-coenzyme A (CoA) pathway, which is the main carbohydrate-driven pathway [42]. The conversion of acetyl-CoA to butyrate is essential to bacteria growth [42]. Butyrate is the main source of energy for colon cells [42,43,44]. In our study, we only determined that the lipid biosynthesis pathway was enriched in gut microbiota from patients with HbA1c < 53 mmol/mol (7%) compared to individuals with HbA1c ≥ 53 mmol/mol (7%). Our finding that family *Ruminococcaceae* was enriched in a group of patients with HbA1c < 53 mmol/mol (7%) could support the thesis that this pathway could be enriched in this group as some butyrate-producers belong to *Ruminococcaceae.*

It should be highlighted that associations should be considered as hypothesis-generating findings that should be investigated in further studies. Our observations indicate the importance of exploring the metabolic pathways of gut microbiota according to the metabolic control, and the importance of further studies on associations between metabolic control and microbiota in patients with T1DM.

One could speculate that increased glucose level favors bacteria with some metabolic pathways are responsible for carbohydrates metabolism. Also, poor metabolic control can play a role in shifting for some metabolic pathways. There may also be, independently, a two-way relationship-worse metabolic control that may change microbiota structure and that may hinder a return to better metabolic control, or that microbiota dysbiosis can worsen the metabolic control in patients with T1DM. Further studies are needed to explore it, because we were not able to answer this question fully as it was not the aim of our study.

Some study limitations should be acknowledged. The study population was relatively small. We did not obtain the information about the exact food eaten two weeks before the sample collection; however, the patients completed a survey on commonly consumed products (data not shown). We collected only one stool sample from every patient in a single point time; therefore, we cannot exclude casual changes in time. We did not obtain information about the patients’ antibodies profile at the time of diagnosis and the time of the study. We did not assess HLA alleles in patients. We performed 16S sequencing; however, shotgun sequencing could give information about viruses and fungi, as well as direct information about which metabolic pathways are encoded. In our study, we could only predict KEGG pathways based on functional profiles using PICRUSt. We also had a lack of a healthy control group, which could have made a study more comprehensive. It would also be of value to investigate this issue in the population of T2DM since gut microbiota might modulate glucose homeostasis via different mechanisms in T1DM and T2DM.

## 5. Conclusions

We observed differences in metabolic pathways and alpha diversity in colonic flora according to HbA1c among patients with T1DM. Further studies should be performed to assess the association between metabolic control and microbiota and their metabolic pathways, also regarding autoimmunity profile and HLA alleles, as well as the possible association between the microbiota profile and insulin resistance.

## Figures and Tables

**Table 1:** The baseline characteristics of patients with glycosylated hemoglobin below and equal to or greater than 53 mmol/mol (7%).

Variable	All Patients (*n* = 89)	Patients with HbA1c Below 53 mmol/mol (7%) (*n* = 43)	Patients with HbA1c Equal to or Greater than 53 mmol/mol (7%) (*n* = 46)	*p*-Value
Male sex, *n* (%)	44 (49.4)	24 (55.8)	20 (43.5)	0.29
Age, years	25 (22–29)	26 (23–31)	24 (22–28)	0.16
Duration of diabetes, years	12.5 (7.8–17) ^1^	13 (7–17) ^2^	11.5 (8–16) ^3^	0.75
BMI, kg m^−2^	23.8 (22.1–24.9) ^4^	23.5 (22.3–24.8) ^2^	23.8 (22.1–25) ^5^	0.73
HbA1c, mmol/mol	53 (46–60)	46 (44–50)	60 (56–65)	<0.001
Average glucose level, mg/dl (mmol/l)	154.9 ± 26.2 ^5^ (8.6 ± 1.5)	142.1 ± 21.7 (7.9 ± 1.2)	167.4 ± 24.3 ^5^ (9.3 ± 1.4)	<0.001
Daily carbs, ×10 g	14.4 (10.7–19.2) ^6^	17.1 (11.2–26) ^7^	13.6 (9.4–16) ^8^	0.02
Total insulin dose, IU	44.7 (37.1–55) ^4^	44.7 (36.6–56.6)	44.7 (37.3–54.8) ^4^	0.87
Percentage of basal insulin, %	41 ± 9.6 ^4^	39.5 ± 10.5	42.6 ± 8.5 ^4^	0.14
Daily insulin/body mass ratio, IU/kg	0.65 (0.52–0.77) ^9^	0.67 (0.53–0.77) ^2^	0.62 (0.52–0.75) ^1^	0.97
The amount of glucose measurements per day, *n*/per day	5.9 (4.2–7.9) ^4^	6.8 (5.7–8.5) ^2^	5.1 (4–7.3) ^5^	0.01
Hypothyroidism, *n* (%)	20 (23.8) ^5^	13 (31.7) ^5^	7 (16.3) ^4^	0.16
Celiac disease, *n* (%)	2 (2.3) ^5^	0 (0) ^2^	2 (4.5) ^2^	0.49
Current smoking, *n* (%)	13 (15.5) ^1^	3 (7.1) ^2^	10 (23.8) ^3^	0.07

Continuous variables are summarized as median (interquartile range) or mean (± standard deviation), and qualitative variables are presented as the number (percentages). HbA1c, *glycosylated* hemoglobin; BMI, body mass index. ^1^ Missing in 5 patients; ^2^ missing in 1 patient; ^3^ missing in 4 patients; ^4^ missing in 3 patients; ^5^ missing in 2 patients; ^6^ missing in 19 patients; ^7^ missing in 8 patients; ^8^ missing in 11 patients; ^9^ missing in 6 patients.

**Table 2:** The comparison of eating habits between patients with glycosylated hemoglobin below and equal to or greater than 53 mmol/mol (7%).

Variable	All Patients (*n* = 79) ^1^	Patients with HbA1c Below 53 mmol/mol (7%) (*n* = 40)	Patients with HbA1c Equal to or Greater than 53 mmol/mol (7%) (*n* = 39)	*p*-Value
The consumption of 4 and more meals per day, *n* (%)	64 (81)	33 (82.5)	31 (79.5)	0.78
Eating fruit at least once a day, *n* (%)	58 (73.4)	30 (75)	28 (71.8)	0.80
Eating vegetables at least once a day, *n* (%)	47 (59.5)	21 (52.5)	26 (66.7)	0.25
Snacking maximum 2 times a week between meals, *n* (%)	76 (96.2)	37 (92.5)	39 (100)	0.24
Drinking sweetened beverages or energy drinks, *n* (%)	42 (53.2)	19 (47.5)	23 (59)	0.37
Drinking alcohol less than 2 times a week or no drinking alcohol, *n* (%)	63 (79.7)	33 (82.5)	30 (76.9)	0.57

^1^ 10 answers missing out of 89 patients.

## Data Availability

The data presented in this study are available on request from the corresponding author.

## Supplementary Materials

The following are available online at https://www.mdpi.com/2076-2607/9/1/155/s1, Supplement 1. Taxonomic information about overlaps between groups.

## References

[1] Katsarou A., Gudbjörnsdottir S., Rawshani A., Dabelea D., Bonifacio E., Anderson B.J., Jacobsen L.M., Schatz D.A., Lernmark A. (2017). Type 1 diabetes mellitus. Nat. Rev. Dis. Prim..

[2] Mishra S.P., Wang S., Nagpal R., Miller B., Singh R., Taraphder S., Yadav H. (2019). Probiotics and Prebiotics for the Amelioration of Type 1 Diabetes: Present and Future Perspectives. Microorganisms.

[3] Chervonsky A. (2009). Innate receptors and microbes in induction of autoimmunity. Curr. Opin. Immunol..

[4] Needell J.C., Zipris D. (2016). The Role of the Intestinal Microbiome in Type 1 Diabetes Pathogenesis. Curr. Diabetes Rep..

[5] Davis-Richardson A.G., Triplett E.W. (2015). A model for the role of gut bacteria in the development of autoimmunity for type 1 diabetes. Diabetologia.

[6] Siljander H., Honkanen J., Knip M. (2019). Microbiome and type 1 diabetes. EBioMedicine.

[7] Alkanani A.K., Hara N., Gottlieb P.A., Ir D., Robertson C.E., Wagner B.D., Frank D.N., Zipris D. (2015). Alterations in intestinal microbiota correlate with susceptibility to type 1 diabetes. Diabetes.

[8] De Groot P.F., Belzer C., Aydin Ö., Levin E., Levels J.H., Aalvink S., Boot F., Holleman F., van Raalte D.H., Scheithauer T.P. (2017). Distinct fecal and oral microbiota composition in human type 1 diabetes, an observational study. PLoS ONE.

[9] Soyucen E., Gulcan A., Aktuglu-Zeybek A.C., Onal H., Kiykim E., Aydin A. (2014). Differences in the gut microbiota of healthy children and those with type 1 diabetes. Pediatr. Int..

[10] De Goffau M.C., Fuentes S., Van Den Bogert B., Honkanen H., De Vos W.M., Welling G.W., Hyöty H., Harmsen H.J.M. (2014). Aberrant gut microbiota composition at the onset of type 1 diabetes in young children. Diabetologia.

[11] Qi C.J., Zhang Q., Yu M., Xu J.P., Zheng J., Wang T., Xiao X.H. (2016). Imbalance of fecal microbiota at newly diagnosed type 1 diabetes in Chinese children. Chin. Med. J..

[12] De Goffau M.C., Luopajärvi K., Knip M., Ilonen J., Ruohtula T., Härkönen T., Orivuori L., Hakala S., Welling G.W., Harmsen H.J. (2013). Fecal microbiota composition differs between children with β-cell autoimmunity and those without. Diabetes.

[13] Brown C.T., Davis-Richardson A.G., Giongo A., Gano K.A., Crabb D.B., Mukherjee N., Casella G., Drew J.C., Ilonen J., Knip M. (2011). Gut microbiome metagenomics analysis suggests a functional model for the development of autoimmunity for type 1 diabetes. PLoS ONE.

[14] Vatanen T., Franzosa E.A., Schwager R., Tripathi S., Arthur T.D., Vehik K., Lernmark Å., Hagopian W.A., Rewers M.J., She J.X. (2018). The human gut microbiome in early-onset type 1 diabetes from the TEDDY study. Nature.

[15] Gomes J.M.G., Costa J.A., Alfenas R.C.G. (2017). Metabolic endotoxemia and diabetes mellitus: A systematic review. Metabolism.

[16] Brar P.C., Kohn B. (2019). Use of the microbiome in the management of children with type 2 diabetes mellitus. Curr. Opin. Pediatrics.

[17] Sikalidis A.K., Maykish A. (2020). The Gut Microbiome and Type 2 Diabetes Mellitus: Discussing A Complex Relationship. Biomedicines.

[18] Tao Y.W., Gu Y.L., Mao X.Q., Zhang L., Pei Y.F. (2020). Effects of probiotics on type II diabetes mellitus: A meta-analysis. J. Transl. Med..

[19] Ho J., Reimer R.A., Doulla M., Huang C. (2016). Effect of prebiotic intake on gut microbiota, intestinal permeability and glycemic control in children with type 1 diabetes: Study protocol for a randomized controlled trial. Trials.

[20] Diabetes Care (2020). 6. Glycemic Targets: Standards of Medical Care in Diabetes 2020. Diabetes Care.

[21] Araszkiewicz A., Bandurska-Stankiewicz E., Budzyński A., Cypryk K., Czech A., Czupryniak L., Drzewoski J., Dzida G., Dziedzic T., Franek E. (2019). 2019 Guidelines on the management of diabetic patients. A position of Diabetes Poland. Clin Diabet..

[22] Mrozinska S., Radkowski P., Gosiewski T., Szopa M., Bulanda M., Ludwig-Galezowska A.H., Morawska I., Sroka-Oleksiak A., Matejko B., Kapusta P. (2016). Qualitative Parameters of the Colonic Flora in Patients with HNF1A-MODY Are Different from Those Observed in Type 2 Diabetes Mellitus. J. Diabetes Res..

[23] Bolyen E., Rideout J.R., Dillon M.R., Bokulich N.A., Abnet C.C., Al-Ghalith G.A., Alexander H., Alm E.J., Arumugam M., Asnicar F. (2019). Reproducible, interactive, scalable and extensible microbiome data science using QIIME 2. Nat. Biotechnol..

[24] Martin M. (2011). Cutadapt removes adapter sequences from high-throughput sequencing reads. EMBnet. J..

[25] Callahan B.J., McMurdie P.J., Rosen M.J., Han A.W., Johnson A.J.A., Holmes S.P. (2016). DADA2, High- resolution sample inference from Illumina amplicon data. Nat. Methods.

[26] Rognes T., Flouri T., Nichols B., Quince C., Mahé F. (2016). VSEARCH: A versatile open source tool for metagenomics. PeerJ.

[27] Janssen S., McDonald D., Gonzalez A., Navas-Molina J.A., Jiang L., Xu Z.Z., Winker K., Kado D.M., Orwoll E., Manary M. (2018). Phylogenetic placement of exact amplicon sequences improves associations with clinical information. Msystems.

[28] Vázquez-Baeza Y., Pirrung M., Gonzalez A., Knight R. (2013). EMPeror: A tool for visualizing high-throughput microbial community data. Gigascience.

[29] Pedregosa F., Varoquaux G., Gramfort A., Michel V., Thirion B., Grisel O., Blondel M., Prettenhofer P., Weiss R., Dubourg V. (2011). Scikit-learn: Machine learning in python. J. Mach. Learn. Res..

[30] Mandal S., Van Treuren W., White R.A., Eggesbø M., Knight R., Peddada S.D. (2015). Analysis of composition of microbiomes: A novel method for studying microbial composition. Microb. Ecol. Health Dis..

[31] Mallick H., Tickle T.L., McIver L.J., Rahnavard G., Nguyen L.H., Weingart G., Ma S., Ren B., Schwager E., Subramanian A. Multivariable Association in Population-Scale Meta’omic Surveys. https://huttenhower.sph.harvard.edu/maaslin2/.

[32] Segata N., Izard J., Waldron L., Gevers D., Miropolsky L., Garrett W.S., Huttenhower C. (2011). Metagenomic biomarker discovery and explanation. Genome Biol..

[33] Kanehisa M., Goto S., Sato Y., Furumichi M., Tanabe M. (2012). KEGG for integration and interpretation of large-scale molecular data sets. Nucleic Acids Res..

[34] Langille M.G.I., Zaneveld J., Caporaso J.G., McDonald D., Knights D., Reyes J.A., Clemente J.C., Burkepile D.E., Thurber R.L.V., Knight R. (2013). Predictive functional profiling of microbial communities using 16S rRNA marker gene sequences. Nat. Biotechnol..

[35] R Core Team (2016). R: A Language and Environment for Statistical Computing.

[36] Kostic A.D., Gevers D., Siljander H., Vatanen T., Hyötyläinen T., Hämäläinen A.M., Peet A., Tillmann V., Pöhö P., Mattila I. (2015). The dynamics of the human infant gut microbiome in development and in progression toward type 1 diabetes. Cell Host Microbe.

[37] Gaike A.H., Paul D., Bhute S., Dhotre D.P., Pande P., Upadhyaya S., Reddy Y., Sampath R., Ghosh D., Chandraprabha D. (2020). The Gut Microbial Diversity of Newly Diagnosed Diabetics but Not of Prediabetics Is Significantly Different from That of Healthy Nondiabetics. mSystems.

[38] Castaner O., Goday A., Park Y.M., Lee S.H., Magkos F., Shiow S., Schröder H. (2018). The Gut Microbiome Profile in Obesity: A Systematic Review. Int. J. Endocrinol..

[39] Kasai C., Sugimoto K., Moritani I., Tanaka J., Oya Y., Inoue H., Tameda M., Shiraki K., Ito M., Takei Y. (2015). Comparison of the gut microbiota composition between obese and non-obese individuals in a Japanese population, as analyzed by terminal restriction fragment length polymorphism and next-generation sequencing. BMC Gastroenterol..

[40] Huang Y., Li S.C., Hu J., Bin Ruan H., Guo H.M., Zhang H.H., Wang X., Pei Y.F., Pan Y., Fang C. (2018). Gut microbiota profiling in Han Chinese with type 1 diabetes. Diabetes Res. Clin. Pract..

[41] Fassatoui M., Lopez-Siles M., Díaz-Rizzolo D.A., Jmel H., Naouali C., Abdessalem G., Chikhaoui A., Nadal B., Jamoussi H., Abid A. (2010). Gut microbiota imbalances in Tunisian participants with type 1 and type 2 diabetes mellitus. Biosci. Rep..

[42] Vital M., Karch A., Pieper D.H. (2017). Colonic Butyrate-Producing Communities in Humans: An Overview Using Omics Data. mSystems.

[43] Kim C.H. (2018). Microbiota or short-chain fatty acids: Which regulates diabetes?. Cell. Mol. Immunol..

[44] Liu H., Wang J., He T., Becker S., Zhang G., Li D., Ma X. (2018). Butyrate: A Double-Edged Sword for Health?. Adv Nutr..

